# COVID-19 and the Nervous System from a Cuban Experience

**DOI:** 10.3390/bs13090776

**Published:** 2023-09-17

**Authors:** Maria de los Angeles Robinson-Agramonte, Teresa Serrano Sánchez, Elena Noris García, Orlando Rafael Serrano Barrera, Dario Siniscalco

**Affiliations:** 1 International Center for Neurological Restoration, Neuroimmunology Department, University of Medical Sciences of Havana, Havana 11300, Cuba; terresaserrano793@gmail.com; 2 Institute of Nephrology, Department of Immunology, Havana 10600, Cuba; anoris@infomed.sld.cu; 3 “Dr. Ernesto Guevara de la Serna” General Hospital, Las Tunas University of Medical Sciences, Las Tunas 75100, Cuba; orlandosb@gmail.com; 4 Department of Experimental Medicine, Division of Molecular Biology, Biotechnology and Histology, University of Campania, 80138 Naples, Italy

**Keywords:** COVID-19, nervous system, neuroinflammation, SARS-CoV-2, neuropsychiatric disorders, psychoneuroimmunology

## Abstract

Neuropsychiatric manifestations of viral infections (both per se and secondary to the neuroinflammatory reaction of the host) are mainly attributed to immunological reactions, so many aspects of their pathogenesis are still nuclear. Some novel therapeutic strategies are progressively emerging in which a vaccination may be having a particular impact on recovery and reduction of death. In this context, it is accepted that the SARS-CoV-2 virus is profoundly neurotropic and neuroinvasive, with various effects on the nervous system, although there is no complete understanding of the mechanism of neuroinvasion, brain injury, or short- or long-term neuropsychiatric sequelae. Therefore, it is necessary to understand the post-infectious manifestations of COVID-19 to guide the management of neuropsychiatric diseases. Thus, based on different research groups focused on this field, in this manuscript we summarize papers on COVID-19 and the nervous system (NS) published in a series of articles by Cuban authors. This review focuses on cognitive and affective emotional states, pathogenesis, biomarkers, clinical manifestations, and intervention strategies.

## 1. Introduction

Severe acute respiratory syndrome coronavirus 2 (SARS-CoV-2) is a coronavirus responsible for the coronavirus disease 2019 (COVID-19) pandemic. The disease caused by this new coronavirus is characterized by an extremely wide variety of clinical manifestations and a complex pathogenesis with very high mortality. The loss of smell and taste is highly recommended as evidence of potential SARS-CoV-2 virus carriers, as well as a sign that the central nervous system (CNS) is involved in SARS-CoV-2 infection.

Since the declaration of the COVID-19 pandemic, several case reports of both the peripheral and the central nervous system have been published. The association between CNS demyelination and viral infection has also long been documented and reported following SARS-CoV-2 infection. In addition, a growing body of literature shows a probable association between SARS-CoV-2 infection and the development of different types of CNS manifestation. Although causality cannot readily be inferred, this review may call attention to the general impact of SARS-CoV-2 infection on nervous system and the necessity of translational focusing of attention to these patients; it considers not only the causal relationship through a para- or post-infectious immune-mediated etiology in COVID-19 patients but also the neurobiological aspects involving the disease course in patients with and without CNS disease history. In addition, this article forms a point of view regarding the psychological and social impact of the pandemic, such as in the region of Cuba. This relationship needs to be clarified in future research.

Therefore, the aim of this review, the first of this series, is to describe the most relevant data about the neurological manifestation and COVID-19 that have emerged from the experience of Cuban authors and been published in papers between 2020 and 2022.

## 2. Methods

### 2.1. Search Strategy

For this narrative review, we searched Medline/PubMed, Google Scholar and Web of Science, and Cochrane and Ovid^®^. In all cases, the searches were conducted for papers in all languages published from 1 January 2020 to 31 December 2022 using MESH terms and free text words for the following search keys: “SARS-CoV-2” OR “COVID-19” AND “psychological safety”, “social isolation”, “CNS”, “neuroprotection”, “neuroinvasion”, “biomarkers”, “cognition”, “affective disorders”, “clinical manifestation”, “brain lesion”, “risk management”, “neuroimmunology”, “psychoneuroimmunology”, and “therapeutic strategy”.

### 2.2. Inclusion and Exclusion Criteria

**Figure 1:**
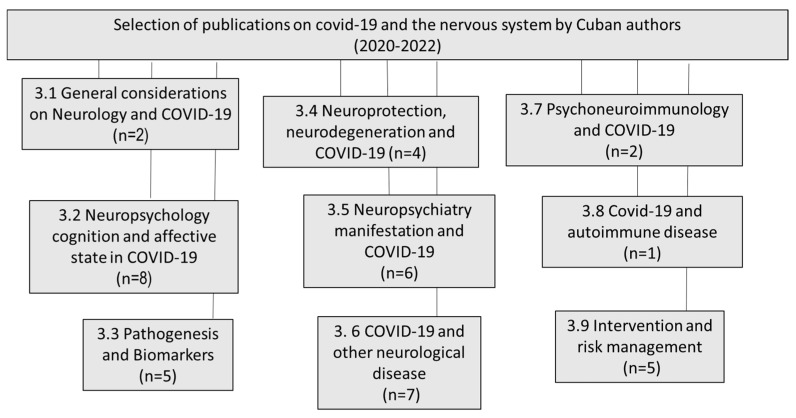
Distribution by topic of the papers published by Cuban authors on COVID-19 and the nervous system (2020–2022).

We reviewed all of the articles published in the English and Spanish languages and selected the most relevant ones, excluding chapter books and not-indexed papers. Figure 1 shows the scientific production of Cuban authors in the period of 2020–2022 and the main topics.

Papers from Cuban authors were searched for in all languages, but only articles in English and Spanish were found.

### 2.3. Data Collection and Extraction

**Figure 2:**
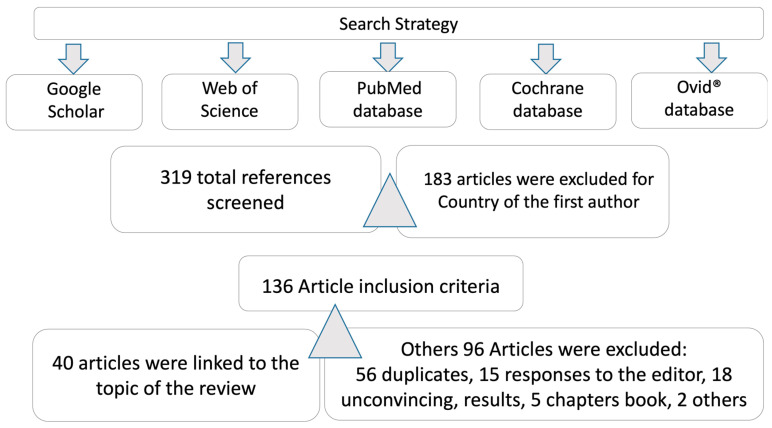
Search strategy and databases reviewed.

Firstly, two authors searched and screened the Medline/PubMed, Google Scholar, and Web of Science databases for relevant titles and abstracts. Full text articles and related references were subsequently searched and analyzed. Debates on inclusion/exclusion criteria were resolved by team consensus. A final analysis included the Cochrane and Ovid^®^ website. Articles published on COVID-19 and the nervous system (NS) were selected, not excluding review papers (Figure 2).

## 3. Results

### 3.1. General Considerations on Neurology and COVID-19

#### 3.1.1. Neurologists and COVID-19

Patients with COVID-19 and impaired consciousness should receive appropriate neurological care and undergo neurological examinations, including neuroimaging, electroencephalogram, and cerebrospinal fluid studies, where appropriate. An article by Josephson [1] pointed out that we must not only rely on technology but also on the thinking of neurologists to solve the pandemic. As the authors pointed out, “[T] his is a moment of courage, empathy, self-care and teamwork. It is also a time of confusion and overwhelming emotion. I encourage you to use your basic neurology skills (reflective listening and problem solving) and walk with humble confidence as you take your immediate next step and the next. Let us unite now, for the sake of our patients and our own humanity” [1].

#### 3.1.2. COVID-19-Induced Brain Dysfunction

SARS-CoV-2 mainly affects the respiratory system, but the damage caused by this virus also extends to other systems, including the CNS, and the neurological mechanisms of infection can be direct or indirect. In their article, Valdés-Sosa et al. [2] analyzed the mechanisms that affect the brain, indicating that the convergence of causal agents of neuronal, psychological, and social damage affects the complexity of the brain. The authors also raised the need to develop less expensive and more accessible methods for low-income populations, showing different methods of the study and diagnosis of brain dysfunctions in patients affected by COVID-19. The authors concluded that access to technology is essential to alleviate growing inequalities in a world where aging and pandemics are a growing risk factor.

### 3.2. Neuropsychology, Cognition, and Affective State in COVID-19

#### Psychological Security and COVID-19

The COVID-19 pandemic has been a challenge for healthcare systems around the world [3]. The effects on mental health run parallel to the pandemic and occur in both healthy and sick people, and it is estimated that these effects will be among the most serious sequelae of the disease. Some reports referred to an underestimation of scientific production on the management of mental health within programs to combat the disease throughout the world and mentioned that the population is prioritized through actions based on spontaneity [4,5]. Recent studies published by Gutiérrez Álvarez AK et al. and Broche-Pérez Y [6,7], based on a systematic review between December 2019 and April 2020, searched 7346 articles and documents on COVID-19. In total, 36 articles were descriptive of “psychological safety”, “emergency healthcare for people in a pandemic”, and “social isolation”, and 11 articles were related to mental health and psychological safety [7]. The results showed a lack of a psychological safety protocol in the population at greatest risk and the absence of a psychological care protocol for people working directly with patients suffering from COVID-19.

On the other hand, the published protocols only argued the concept of psychological safety developed by Amy Edmondson et al. The previous definition was based on building a solid organization under the belief that the environment is safe, thus guaranteeing the resilience of people at risk. Physical care, a fundamental premise for this type of intervention, is necessary to obtain a more effective result, without any type of non-verbal communication, both in first aid and in crisis. As is known, the crisis proposes basic principles for remote psychological care (immediacy, management of expectations, simplicity of action and experience in a unique experience) [7,8,9], with the psychological first aid strategy providing psychologists with an immediate perspective to alleviate patients’ state of mind (listening, facilitating verbal expression and emotions) [7].

Under the aforementioned bases, protocols were implemented to support and guide specific actions for the psychological safety of health persons at risk of COVID-19. This was based on the best experiences published during the current pandemic from psychological intervention groups from China, Italy, Spain, and Peru [10], which provided psychologists with a validated intervention tool and a psychological safety guide.

In their article, Martinez-Perez and collaborators noticed that adverse outcomes for mental health in confinement and epidemic situations are not usually evident; therefore, a systematic approach is required for the identification of cases and the selection of appropriate interventions. From this viewpoint, they characterized the aspects of affective emotional states and their relationship with some socio-demographic factors in an adult population of Puerto Padre, an urbane locality in a eastern area of the country, as a consequence of the context generated by COVID-19. The study, carried out with 206 subjects over 18 years of age, covered emotional affective states like stress, anxiety, and depression from the DASS-21 scale applied to the subjects. As result, 32.52% of the studied subjects presented at least one of the three studied emotional states; the three affective conditions showed a significant relationship with history of mental illness [11]. These results, published from Cuba, agree with other studies carried out and published by other authors abroad that found high levels of depression (43.3%), anxiety (45.4%), and turnover for post-traumatic stress (31.8%) [12]. In summary, the authors referred to a high index of those three emotional affective states in the population studied, of which the light and moderate forms prevailed. The biggest associated factor was having a single antecedent of mental illness, and the higher correlation between stress and anxiety had the most relevant impact.

Callís-Fernández and collaborators also published an article describing the high contagiousness of COVID-19, the growth of confirmed cases and deaths in the world, and social isolation as causes of the origin of negative emotions and thoughts spreading, threatening the mental health of the population in a study carried out at the Josué País García Polyclinic in Santiago de Cuba province. The paper, a descriptive, cross-sectional study that was carried out between April and May 2020, included 154 single senior citizens, who were subjected to a semi-structured interview including the listed variables (age, sex, marital status, cohabitation, occupation, comorbidity, anxiety, depression, irritability, stress). The results showed that more than 55% of patients belonged to the female sex, 56.5% were older than 70 years, and more than 85% had pathologies considered risky for COVID-19. In addition, the authors found that a normal level of irritability prevailed, and mild levels of anxiety and depression were observed in the studied subjects. However, more than 80% of the subjects were found to suffer from stress [13]. A conclusion from this analysis is that social isolation, as a measure to avoid contagion, had a hard impact on the mental health of senior citizens.

Similar studies performed in Pinar del Rio province were focused on the relevant impact of depressive symptoms in patients diagnosed with COVID-19 throughout the course and recovery of the disease, as well as on a more effective response to treatment [14,15].

The last article published by Zayas-Fajardo et al. focused on the psychological impact of social isolation due to COVID-19 on children, adolescents, and family. In this paper, the authors defined the importance of preventive social isolation due to COVID-19 on children and adolescents and characterized the aspects of the psychological impact of social isolation on these populations and on their families. This study was carried out at the Jimmy Hirzel Polyclinic of Bayamo, located in a Cuban province, as a cross-sectional descriptive and observational investigation and belonging to the aforementioned health district. All subjects were also interviewed following the FF-SIL test. At the time, this research was useful because it revealed the highest psychological repercussions observed in children from 5 to 9 years old (48.8%), with a history of psychiatric disorders like attention deficit disorder and neurotic disorders. In the same line of events, anxiety, motor disturbance, verbal hyperactivity, fear of death, and somatization of anxiety were the most frequent psychological manifestations observed in children and adolescents, whereas in their families, these symptoms were more related to anxiety, fear, and depression, especially in mothers [16]. Finally, a study published by Espinosa Ferro defined the importance of sanitary protection and social isolation due to COVID-19 in medicine students, and they focused on the repercussions of isolation in this population, which agrees with the results obtained by other Cuban authors [14,15,17].

### 3.3. Pathogenesis and Biomarkers

Respiratory distress syndrome is an acute and diffuse lung disease that causes hypoxemia, decreased lung compliance, and increased dead space. Patients with COVID-19 are known to develop this syndrome. Calixto Machado, in his article related to this topic, raised the need to know whether this neurological condition could partly explain the respiratory distress that these patients commonly present, suggesting a brain stem dysfunction as a possible cause. This suggests the need for more specific and aggressive treatments with the direct participation of intensive care providers and neurologists [18]. In another article, Machado indicated that the angiotensin-converting enzyme 2 (ACE2) receptors that bind to SARS-CoV-2 are widely distributed not only in the respiratory system but also in other systems. These receptors are also expressed in glial cells and neurons, both of which are targets of SARS-CoV-2 [19,20]. Another example of alteration in the NS in patients with SARS-CoV-2 is detailed in the article by Padrón González and Dorta Contreras, where it is stated that the virus not only attacks the CNS but also the peripheral NS, producing manifestations that can be mild and transitory. However, complications of direct cytopathic damage or complications secondary to the effects of the inflammatory response can arise [21]. In line with the study of biomarkers and the pathogenesis of SARS-CoV-2, Royer Rodriguez-Guzmán, in a letter to the editor in the journal *Rev Neurol. 2020*, highlighted the interesting results of Redondo Urda describing SARS-CoV-2 as the causative agent of Guillain Barré syndrome (GBS), one of the complications caused by this virus [22,23]. It should be noted that although the authors were able to diagnose these patients using different tests, it would be necessary, given that this pathology is immune-mediated, to perform cytological albumin dissociation studies. These authors believe that to support the diagnosis it would be necessary to perform a Reibergram, a tool that allows the functional state of the blood–brain barrier (BBB) to be understood [24,25]. In these patients, there is a severe involvement of the endothelial cells of the BBB and this is an important factor in the spread of the virus to the cerebrospinal fluid and subsequently into the brain tissue [22].

### 3.4. Neuroprotection, Neurodegeneration, and COVID-19

The neuro-inflammatory base of various neurological processes, as well as that the nervous system becomes a blank of neuroinflammation to induce and/or contribute to neurodegenerative disease progression, is well known. Another important aspect is related to the coinciding of alarm signs or prodrome symptoms of neurodegenerative disorders with COVID-19-specific symptomatology, like anosmia and ageusia in Parkinson’s disease (PD). On the other hand, with this disease, the stress derived from the pandemic could also lead to a less effective response to treatment, causing an apparent worsening of the diagnosis due to the depletion of the compensatory mechanism. Therefore, it is important to create a development strategy for neuroprotection in the context of COVID-19 to reduce the uncontrolled neuroinflammation occurring in the pandemic and to reduce the neurological impact of infection in asymptomatic patients who are positive for SARS-CoV-2.

The term “neuroprotection” refers to the protective effect of any substance, chemical, or biological molecule with effects on the CNS to prevent, mitigate, or delay the neurodegenerative process typical of diseases such as Alzheimer’s, PD, and brain injuries and to prevent neuronal death by apoptosis or other pathways of neurodegeneration. Direct and indirect effects on mental health, including not only neurological events but also neuropsychiatric and neuropsychological manifestations, either early on or in the long term, have been associated with COVID- 19, and it is believed that in the future these disorders, in particular, neurodegenerative disorders, will increase around the world. Diverse theories, some of which were mentioned previously in this manuscript, point to brain damage as a consequence of direct neuronal infection by SARS-CoV-2 that contributes to neurodegenerative disorders in the long term [21]. However, the exact mechanism has not been found yet.

Currently, there is widespread interest in the application of neuroprotectants to prevent and treat many CNS diseases, such as Alzheimer’s disease, Parkinson’s disease, schizophrenia, stroke, and concussion-derived syndromes.

NeuroEPO is a neuroprotective drug widely used in neurodegenerative diseases. A recent study by Julio C. García et al. [26] indicated that this molecule is capable of potentiating the endogenous defenses of the brain, which could prove to be effective in locally diminishing the immediate and possibly long-term damage of the virus on the CNS.

In a letter to the editor, Lozada et al. [27] commented on the article by Mejides et al. [28], concluding that more studies are needed on the relationship between COVID-19 and neuroimmunology, taking into account that there is evidence that SARS-CoV-2 has a tropism for the CNS.

Taking these aspects into account, authors from different regions of the world have disclosed valuable information on some molecules that could have a protective effect against neuroinflammation due to their immunoregulatory activity. Romero et al. indicated that melatonin has the ability to restore the homeostasis of the BBB, limiting microvascular hyperpermeability [29]. Another molecule proposed by Xu et al. is vitamin D [30]. The type II pneumocyte is the main target of SARS-CoV-2 infection, and it has been shown that vitamin D decreases the degree of apoptosis and stimulates the synthesis of surfactant in these cells, which creates a favorable environment against lung damage. On the other hand, vitamin D induces the regulation of neurotrophic factors, essential proteins involved in the survival, differentiation, and maintenance of nerve cells, both centrally and peripherally [30]. The flavonoid luteolin, a polyphenol that has demonstrated anti-inflammatory, antioxidant, anticancer, and cytoprotective activity, is another component that has been studied to assess its neuroprotective effect. Kempuraj D et al. [31] indicated that luteolin, through its role in the suppression of the hyperactivation of the immune cells, astrocytes, and neuroglia and the modulation of oxidative stress, could exhibit a neuroprotective effect against COVID-19 by maintaining the integrity of the BBB and preventing tissue damage.

In their letter, Lozada et al. concluded that the evidence is not enough to be able to affirm with total certainty that these agents could serve as effective and safe therapy [27]. Future research will allow for the evaluation of the capacity of these molecules in the prevention of neurological complications of COVID-19.

C-phycocyanin has shown protective activity in animal models of diverse human health diseases, often reflecting antioxidant and anti-inflammatory effects. The beneficial effects of C-phycocyanin seem likely to be primarily attributable to its covalently attached chromophore phycocyanobilin (PCB). The authors in this paper reviewed the most recent research in which C-phycocyanin and/or PCB, administered orally, parenterally, or intranasally, achieved marked protective effects in rodent and cell culture models of ischemic stroke and multiple sclerosis, and from this experience they suggested that these agents may likewise be protective against Alzheimer’s disease, Parkinson’s disease, and COVID-19 and its neurological complications [32].

### 3.5. Neuropsychiatry Manifestation and COVID-19

The COVID-19 pandemic represents a challenge for the future in health systems due to its impact outside of the respiratory system. Identifying the main neurological and neuropsychiatric manifestations associated with COVID-19 has been the focus of several research groups around the world, either as reports of cases or papers describing the main clinical manifestations in the short or long term of COVID-19.

Aguiar-González et al. published two papers in 2021 in *Magazine of the Medical University of the Province of Pinar del Río* (www.revgaleno.sld.cu, accessed on 20 July 2023), one of which is titled “Neurological manifestations in patients with COVID-19” [33]. In this work, the authors referred to the challenge that the COVID-19 pandemic represents for health systems due to the impact on different body systems, such as the respiratory, cardiovascular, and nervous systems. From about 30 articles reviewed by these authors, several conclusions were reached: (1) The olfactory nerves and the invasion of the peripheral nerve terminals are the main suggested routes of entry of the SARS-CoV-2 virus into the brain [33], and (2) severe neural symptoms are associated with lesions of other organs such as the CNS and also with cytokine storm. In addition, the authors concluded that headache appears to be the most common neurological manifestation in patients with COVID-19, whereas rhabdomyolysis, acute necrotizing hemorrhagic encephalopathy, Guillain Barré syndrome (GBS), meningitis, and encephalitis are less frequent in COVID-19 patients [34,35,36]. This work described the most frequent neurological symptoms in patients in different age groups and with severe/mild COVID-19, stratified into CNS disorders and peripheral nervous system (PNS) disorders:

#### 3.5.1. Most Frequent Symptom of CNS in COVID-19

Headache was the most common CNS neurological manifestation reported by patients with COVID-19. Between 8 and 34% of patients in China presented this symptom, generally with mild intensity [37]. In addition to headache, vomiting, and nausea, vertigo was a recurring symptom in 7–9% of patients with COVID-19.

Another two papers related to the effect on the CNS were published by Leon et al. [38,39].

The first one described in a general way the high probability that the ventilatory dysfunction of patients is based on the involvement of the cardiorespiratory center in the brain stem beyond lung lesions and why it is very important to be alert to neurological manifestations that can occur even in the early stages of patients with COVID-19. Experimental and clinical data from SARS-CoV-2 infections in the brain were presented to describe the different pathways through which the virus manages to invade the brain via the olfactory CNS to spread locally. The authors also described the trans-synaptic pathway, the pathway that connects the cardiorespiratory center with the mechanoreceptors and chemoreceptors of lung tissue and the respiratory tract, underlining the impact of this pathway on ventilatory failure in these patients. A particular aspect reviewed in this work is the invasion of neurons and neuroglia by coronaviruses, facilitated by the expression of ACE2 on their surface, which allows direct infection at the CNS level parallel to the inflammatory process at the systemic level that occurs during COVID-19. The latter compromises the BBB and provides access of inflammatory elements to the CNS and neuroinflammation, reactive astrogliosis, and microglial activation, which means that various neurological processes can potentially lead to ventilatory disorders based on the influence on the cardiorespiratory center due to the impact on the brainstem. Finally, the authors emphasized the need to be alerted early to the presence of neurological manifestations in patients with COVID-19, as well as the need to be able to count on tools to identify predictive biomarkers and prognoses for intervention and sequelae prevention [38].

The second article, titled “Olfactory dysfunction and COVID-19”, referred to the smell disorders that precede or accompany SARS-CoV-2 infection as a simple clinical manifestation of COVID-19 [39]. This review article aimed to analytically summarize the scientific evidence of the relationship of olfactory dysfunction derived from SARS-CoV-2 infection and was designed by using the Google Scholar search engine with the descriptors “COVID-19”, “SARS-CoV-2”, “anosmia”, and “hyposmia”. The results of the review ranged from case reports to published research and review articles on the involvement of the olfactory pathway in COVID-19 and the possible pathophysiological mechanisms involved. Similar to other previous results discussed here, these authors concluded that olfactory disorders preceding or accompanying SARS-CoV-2 infection could be an isolated clinical manifestation of COVID-19 and therefore should be considered. Based on this last perspective, the authors also considered that quantitative neurophysiological testing and hybrid imaging of the olfactory bulb could be useful tools to clarify the mechanism and establish the probable association between the structural changes and connectivity produced by the invasion of SARS-CoV-2 of the olfactory pathway and suggested adding a history of smell disorders to the clinical history of patients with COVID-19 as mandatory data, since the demonstration of the relationship between smell disorders and SARS-CoV-2 infections could have diagnostic or prognostic value [39].

#### 3.5.2. Most Frequent Symptoms of PNS in COVID-19

Based on this review, the authors considered that anosmia/hyposmia and, secondarily, taste disorders, constitute primary symptoms associated with SARS-CoV-2 infection and that their early identification is of vital importance to reducing the impact of the infection. Although anosmia is not specific to SARS-CoV-2 infection, it is linked to taste disorders, which are common in people with COVID-19, even in the absence of nasal symptoms [40], and could be early markers of SARS-CoV-2 infection. Regarding its origin, the authors stated that it follows direct damage to the olfactory receptor neurons [41]. This last hypothesis is favored by the expression of receptors for ACE 2 proteins and for serine transmembrane protease 2 (TMPRSS2) in cells of the olfactory epithelium, which are necessary for SARS-CoV-2 infection in humans [31,32] and were described in anosmia in 5.1% of patients and in ageusia in 5.6%.

Machado and DeFina also wrote about anosmia and ageusia, arguing that they are the only symptoms prior to COVID-19 [19]. Their article defines the terms “anosmia” as the temporary or permanent loss of the ability to detect one or more odors and “ageusia” as the loss of taste functions of the tongue, mainly including the inability to taste sweetness, acidity, bitterness, salinity, or umami—which is a pleasant/tasty taste—and the loss of olfactory neural pathways, considering that this olfactory conduction pathway begins with olfactory receptors and taste. The article also details how the mechanisms lead to loss of smell and taste from SARS-CoV-2 infection [42]. Regarding this last aspect, the authors emphasized that the loss of smell could be due to inflammation of the nose and paranasal sinuses due to chronic sinusitis, head injuries, or nervous disorders (such as Parkinson’s disease) [43]. In addition, they stated that loss of smell from a viral infection such as the common cold is the second most common cause of loss of smell, present in about 12% of all cases of anosmia, and noted that these episodes usually occur when the virus infects the nose [20]. In this work, the authors also reported that the peripheral trigeminal or olfactory nerves, known routes of penetration of coronaviruses into the CNS, can explain the complications derived from brain invasion by SARS-CoV-2, such as demyelination and mediated autoimmunity by T cells—reactions that may be part of the path of infection spread—so the incidence of dyssomnia and dysgeusia could be a potentially painful consequence of these brain lesions [42]. These authors considered the most relevant signs and symptoms of COVID-19 to be fever (98%), cough (76%), dyspnea (55%), and myalgia or fatigue (44%). However, an association between COVID-19 and altered olfactory and taste functions were suggested, although smell seems to be more affected than taste.

Robinson-Agramonte et al. published a relevant article titled “Impact of SARS-CoV-2 on neuropsychiatric disorders” in 2021 [44] based on the high number of reported case series and unclear evidence of the involvement of the immune system in the pathological mechanism of COVID-19. The authors focused the review on the suggested immunological mechanism involving the direct effect of SARS-CoV-2 infection on the CNS and neuroinflammation and described the neuropsychiatric disorders associated with COVID-19. Symptoms and signs such as depression, anxiety, mood disturbances, psychosis, post-traumatic stress disorder, delirium, and cognitive impairment appeared to be common in COVID-19 survivors, as well as a worsening of scores on psychopathology measures after a history of comorbidities. The authors also made a critical analysis of the innate and adaptive immune system aspects involved in the mental health disorders that occur with COVID-19 [44,45].

Following what has been described in these articles, neuroinflammation impacts disease pathology such as schizophrenia and autism and involves mental illness in COVID-19 in a close interaction between the systemic compartment and the brain [45].

Lorigados and Pavón summarized the main experiences reported in the presence of CNS conditions related to COVID-19 and analyzed the relationship between COVID-19 and neurological diseases [46]. They described the presence of IgM-class anti-CoV antibodies in 12% (*n* = 183) of patients with clinical suspicion of acute encephalitis, different from that reported for prior MERS-CoV infection (26% in patients with mental disorders and 9% in those with a history of seizures). The authors also referred to the behavior of the most frequent neurological manifestations associated with COVID-19 and pointed out that, in a record published by Ahmad I and Rathore FA in 2020, between 68 and 75% of COVID-19-positive patients had loss of smell and between 71 and 43% had dysgeusia. On the other hand, there is recent evidence of CNS invasion in patients with SARS-CoV-2 and with positive RNA in the CSF and on the neuropathological mechanisms that mediate CNS involvement, which are mediated by hypoxic brain damage and associated with immune mechanisms. The first of these mechanisms is a consequence of severe pneumonia, resulting in systemic hypoxia and leading to cerebral hypoxia; the second is due to an inflammatory cytokine storm followed by activation of T lymphocytes, macrophages, and endothelial cells. Interleukin 6 (IL-6) is known to cause vascular involvement, complement activation, and participate in the coagulation cascade, along with intravascular spread and organ damage.

Unlike the articles reviewed so far, this paper described particular behavioral aspects of the neurological manifestations associated with COVID-19, such as Parkinson’s disease, encephalopathies, GBS, epilepsy, and stroke. In all cases, a clinical case was shown and the behavior of the SARS-CoV-2 infection and the disease was described, with clinical and paraclinical evidence [46]. Finally, the authors called attention to prioritizing the evaluation of neurological events present in these patients, both from the clinical and the prognostic point of view, in order to prevent the most severe course of the disease in SARS-CoV-2 patients.

Regarding neuropsychiatric disorders, Bender and colleagues summarized the scientific evidence on the impact of COVID-19 on neuropsychiatric disorders. The search with the Google Scholar engine was rich and the descriptors of “COVID-19”, “SARS-CoV-2”, and “neuropsychiatric manifestations” were used. This paper described the general clinical manifestations of COVID-19 and subacute or chronic psychiatric sequelae in relation to SARS-CoV-2 infection, including depression, anxiety, and stress. Related to other works, the need for care for health personnel and patients with previous mental illness and chronic neurological diseases, in whom symptoms can worsen and even lead to suicide, is emphasized [47]. Long-term psychiatric complications after SARS-CoV-2 infection are currently unknown. In this paper, the authors referred to studies published by Severance et al. M, who found a higher prevalence of antibodies against four strains of HCoV in patients with a recent psychotic episode compared to non-psychiatric controls, which allowed Severance et al. to suggest a possible relationship between CoV infections and psychosis, with potential for its occurrence following SARS-CoV-2 infection. The authors also made a specific reference to the works published by Valdés-Florido et al., referring to the occurrence of psychotic episodes during the first two weeks of quarantine in patients admitted in Seville, Spain, in whom the confirmation of the diagnosis followed the psychotic disorder criteria of the Diagnostic and Statistical Manual of Mental Disorders (DSM-5). In the opinion of the authors, the episodes were triggered by the stress derived from the COVID-19 pandemic, and half of the patients presented severe suicidal behavior on admission [48]. Bender et al. warned, in turn, about the risk presented by the increase in the number of brief reactive psychotic disorders as a result of the COVID-19 pandemic [47].

### 3.6. COVID-19 and Other Neurological Diseases

The involvement of the CNS has been considered since the first cases of COVID-19, and epilepsy, ataxia, and impaired consciousness are among the most frequent neurological manifestations [20].

#### 3.6.1. Epilepsy and COVID-19

In a letter to the editor, Bender et al. reflected on the need to consider the action protocols related to the appearance of epilepsy seizures in COVID-19 based on the first published case associating epileptic seizures with the course of COVID-19 infection. In the opinion of these authors, many viruses may play a role in the development of epileptic seizures, and in this case the cause may be directly related to a primary infection of the CNS [49]. Another paper published by these authors sent a message to give a warning about the importance of monitoring seizures in epileptic patients who are infected with SARS-CoV-2, because it may increase the risk of epileptic seizures, similar to any acute infection, and in turn, the infection can become a triggering factor for crises. They also stressed that it is not the viral infection that can cause epileptic seizures but sepsis, the fever that accompanies it, and sleep deprivation [50]. Both articles also drew attention to the need for adherence of the professionals involved in the treatment of these patients to following the guidelines of the International League Against Epilepsy, which alerts, among other aspects, about the possible interactions of antiepileptic drugs with the drugs used in disease action protocols.

#### 3.6.2. Hereditary Ataxias and COVID-19

Possible pathophysiological implications and recommendations are the topics of a review article by Velazquez et al. [51]. The authors argued how patients with hereditary ataxias constitute a vulnerable group, given the well-known functional disturbance of the immune system, the central disturbances of respiratory control, and the existence of comorbidities that increase the risk of health worsening derived from COVID-19, such as cardiopathies, diabetes mellitus, and cancer, and underlined a series of practical recommendations. The authors briefly summarized the characteristics of the main types of ataxia and the relationship between ataxin-2 and RNA+ virus infections and suggested that loss of biological function in patients could protect them against infections.

Friedreich’s ataxia is an autosomal recessive cerebellar ataxia caused by abnormally long expansions of the repetitive guanine–adenine–adenine (GAA) sequence, which prevents the correct expression of the Friedreich’s ataxia (FRDA) gene. In this disease, the main warning for its management in the case of SARS-CoV-2 infection is that these patients usually have heart disease and diabetes mellitus, two comorbid conditions that aggravate COVID-19 and increase mortality. On the other hand, these patients also present alterations in the regulation of the respiratory activity, mainly mediated by autonomic dysfunction [51]. This element could be disadvantageous for the disease.

Ataxia-telangiectasia, another type of ataxia with an autosomal recessive pattern of inheritance, is produced by localized mutations in the ATM gene (11q22.3) and is characterized by a cerebellar syndrome associated with a combined immunodeficiency that mainly affects the humoral immune response. Several authors considered that these patients should receive greater family and medical attention during the current pandemic, as they present a significant susceptibility to infections [51,52]. In general, the social isolation of these patients may have consequences on their physical and mental health, given their dependence on rehabilitation services and the emotional and social cognition alterations underlying the pandemic. Based on these elements, the authors suggested a series of recommendations for the management of these patients suffering from COVID-19. The recommendations are were made by the International Society for Movement Disorders and Parkinson’s Disease and the Specialized Committee on Neurogenetics belonging to the Chinese Medical Association [51], as well as by expert consensus on the management strategy of patients with hereditary ataxia during the prevention and control of the novel coronavirus pneumonia epidemic [52]: (1) Maintain the usual treatments, such as vitamin therapy, antioxidants, and others, according to the type of ataxia; (2) maintain physical–motor rehabilitation in a systematic way, fundamentally based on coordination, balance, and walking exercises in their own homes; (3) increase sleep hygiene measures to achieve restful sleep that should not exceed 8 h; (4) carry out relaxation strategies, such as meditation or yoga, in addition to associating the above with listening to relaxing music and maintaining a very optimistic state of mind; (5) maintain communication by telephone with specialist doctors, either at the ataxias center or in the health areas, and, in the event of a suspicious respiratory manifestation, go to the family doctor.

#### 3.6.3. Cerebrovascular Disease and COVID-19

Cerebrovascular disease is the topic of a review article by Bender et al. [53]. Cerebrovascular diseases constitute a global health problem due to their incidence, prevalence, and mortality. In this paper, the casuistry and reports of cases that have been described up to the time of writing and an analysis of the possible pathophysiological implication were included. Attention was drawn to the reduction in reported cases of cerebrovascular disease without casuistic support and alerts about its presentation and related risk factors. Relevant considerations of Morelli and collaborators from the Guglielmo da Saliceto Hospital in Piacenza, Milan, Italy, were shown in this work [53]. Ischemic accidents were described in cerebrovascular disorders as having almost disappeared from stroke units. The authors were of the opinion that the significant reduction in currently registered cerebrovascular accidents can be attributed to the fact that fewer people go to the hospital for fear of becoming infected, although this may be true only for mild and non-disabling cases. Brain bleeds are always disabling, and it is impossible to avoid hospitalization with such a serious condition. A hypothesis put forward by the authors refers to the fact that the increased risk of these vascular events could be related to the controversial role attributed to IL-6 in stroke, whose high levels have a negative effect on the volume of the cerebral infarct in the long term [53]. On the contrary, IL-6 has a protective effect in ischemic stroke that helps improve post-stroke angiogenesis. On the other hand, the presence of thrombocytopenia in patients with mild COVID-19 raises the question of whether decreased platelet levels have an impact on the reduction of strokes. The evidence-based answer stated that the burden of persistent chronic infections or past infections appears to be associated with the risk of stroke [53].

González-García et al. also wrote about some published studies related to the frequency of strokes in patients infected with the virus, which is considered to be between 5 and 20% of COVID-19 cases. They reported that, although the pathophysiological mechanisms by which a stroke can occur in these patients are still unknown, cases of SARS-CoV-2 infection associated with a prothrombotic state capable of causing arterial and venous thromboembolism have been described. In addition, it was argued that an exacerbated inflammatory response with recruitment of blood cells and disproportionate secretion of proinflammatory cytokines constitutes the biological basis of this process. In addition, hypoxia and cardioembolic phenomena were proposed as possible mechanisms. However, the conclusive criterion is the essential need to accurately define the pathophysiological mechanisms that link SARS-CoV-2 infection with the occurrence of stroke and the need to apply more specific treatments to avoid future complications [54].

Based on the impact of dementia in Latin American and Caribbean countries (LACs) and vulnerable populations, Ibanez and col. described, using an underscore report, the impact of SARS-CoV-2 on dementia among LACs, the specific strain on health systems devoted to dementia, and the subsequent effect of increasing inequalities among those with dementia in the region, and also called attention to the implementation of best practices for mitigation and containment, which are facing particularly steep challenges in LACs. They also advised on the necessity for a coordinated action plan, including the development of inexpensive mass testing and multilevel regional coordination for dementia care and related actions. Brain health diplomacy should lead to a shared and escalated response across the region, coordinating leadership, and interaction between governments and international multilateral networks [55].

### 3.7. Psychoneuroimmunology and COVID-19

The durability of COVID-19 worldwide and its impact outside the respiratory system makes it necessary to implement programs that allow the patient to be assessed as a biopsychosocial being in the search for better diagnostic and treatment strategies; in this context, psychoneuroimmunology approaches are necessary for the achievement of more humanistic medicine.

From this perspective, Monet and cols. reported on cases that occurred in Santiago de Cuba, a province in the eastern part of the country, after 11 March 2020. Early in the pandemic, attention was focused on the acute morbidity and mortality associated with COVID-19, whereas months later persistent physical and neuropsychiatric squeals were described after SARS-CoV-2 infection. The authors reported that, although residual or persistent neuropsychiatric symptoms are not uncommon in survivors, post-COVID-19 follow-up revealed a mild and/or asymptomatic infection responsible for cognitive impairment, delirium, extreme fatigue, and relevant mood symptoms, probably as result of the interaction between multiple factors: the nervous, endocrine, and immune systems, with the last one also involving a visceral stress response, with an impact on mental health, favoring the psychological manifestations linked to the severity of the somatic and psychiatric symptoms [56].

Noris and cols, in their article on neuroimmunology and COVID-19, stated that COVID-19 causes psychiatric and somatic disorders as result of the interaction between the nervous, endocrine, and immune systems, which impacts homeostasis. The functioning of the immune system involves a visceral stress response that has an impact on mental health and SARS-CoV-2 that is capable of invading the central nervous system and producing neuroinflammation, favoring the psychological manifestations reported by some authors that are linked to the severity of somatic and psychiatric symptoms. On the other hand, stress, the main mediator of the hypothalamic–pituitary–adrenal (HPA) axis, in the course of the SARS-CoV-2 pandemic produces elevated cortisol levels, which have an immunosuppressive effect, resulting in an imbalance of cytokine production that leads to an immunosuppressive mechanism and increased susceptibility to COVID-19 viral infection [57]. Based on this experience, the authors supported the need to consider the functional interaction of these three systems as the basis for the prevention of neuropsychiatric sequelae produced by SARS-CoV-2.

The neuropsychiatric manifestations of COVID-19 have an impact in the context of this pandemic due to their frequency and based on the clinical status of the patients. In this aspect, the authors referred in this article to the impact of comorbidities and the appearance of neuropsychiatric manifestations and followed the idea of comorbid states, such as immunodeficiencies, obesity, and autoimmune diseases, showing association with cognitive deterioration and Alzheimer’s disease, as well as with more severe forms of the disease. These states can also give rise to immunodeficiency due to their action on the hypothalamus–pituitary–adrenal axis and to the increase in cortisol levels. In addition, increased levels of cytokines (IL-6, IL-4, IL-10, and TNF-alpha) and proteins associated with CNS damage and neurotransmitters (epinephrine and serotonin) have been observed in psychiatric conditions such as schizophrenia and depression, which reinforces the role of the neuroendocrine axis as a relevant hypothesis in the occurrence of these disorders related to SARS-CoV-2 infection [55,57]. Similarly, increased levels of some biomarkers associated with CNS damage, such as the S100 B protein (protein calcium-binding B) and fibroglial acid protein (GFAP) have been observed during these post-COVID-19 neuropsychiatric manifestations [58].

### 3.8. COVID-19 and Autoimmune Diseases

**Figure 3:**
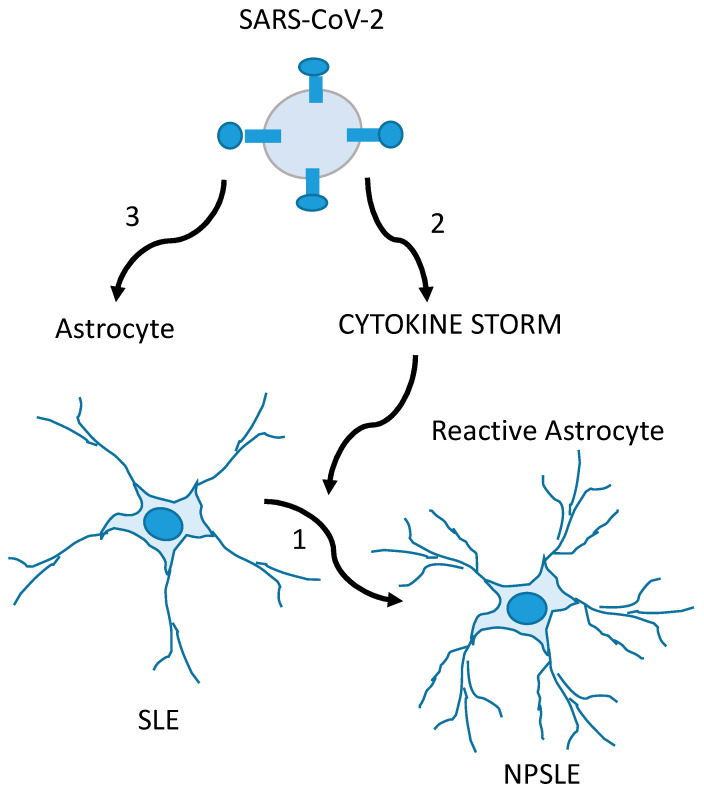
Glia-mediated neuropsychiatric manifestations in SLE (NPSLE) patients with COVID-19 are possibly caused by the CNS involvement of SLE and exacerbated by SARS-CoV-2. In the first mechanism, SLE triggers inflammatory events on normal-functioning astrocytes in the CNS, producing the glial hyper-reactivity (increase in GFAP levels and S100B secretion) found in NPSLE (1). In the second condition, the SARS-CoV-2-induced cytokine storm (together with hypoxemia and thrombotic events) causes glial reactivity, overlapping or not with mechanism 1 (2). In the third mechanism, SARS-CoV-2 is the direct cause of the glial reactivity, overlapping or not with mechanism 1 (3). Adapted from [58], with permission from Elsevier (license # 5586971171821).

Based on the experiences published by Noris and col, the authors emphasized that the best way to approach post-COVID-19 neuropsychiatric disease is a comprehensive and holistic approach. They considered that the latter helps to reduce the associated factors related not only to the possibility of contagion but also to the development of emotional manifestations that lead to greater vulnerability to contraction and to short- and long-term repercussions of the disease. Among the sequelae of SARS-CoV-2 infection, autoimmune diseases related to frequency of appearance and crises stand out [59]. In this sense, systemic lupus erythematosus (SLE) deserves special interest: Neuropsychiatric manifestations in patients with COVID-19 may be the result of CNS damage, aggravated by infection by this neurotrophic virus and/or by the ability of the virus to induce the appearance of SLE as a post-COVID-19 neuropsychiatric manifestation. In these cases, a research experience by Noris et al. demonstrated that the quantification of the S100B level is an useful variable in the early identification or diagnosis of these neuropsychiatric manifestations in the course of infection or after damage to the post-COVID-19 CNS [58] (Figure 3).

### 3.9. Intervention and Risk Management

In the COVID-19 pandemic caused by SARS-CoV-2, neurologists have focused their attention on the early identification of suggestive manifestations of the neurological impact of the disease. In this context, they have explored related chronic diseases and the possibility of achieving a more effective understanding of symptoms derived from COVID-19 infection with respect to those derived from the course of pre-existing neurological diseases. Diaz de la Fe and cols reviewed the current state of knowledge of infection with SARS-CoV-2 and the management of the risks in multiple sclerosis (MS), as well as the risks of general comorbidities associated with COVID-19 [59]. The authors reviewed main factors of MS, such as relapses, and the maximum tolerated dose of treatment from clinical and experimental evidence. They showed information on the main condition for assessing the risk for COVID-19 in MS based on the immunocompetence status of patients, especially those using disease-modifying therapies (DMT) [59]. They commented that first-line treatments do not seem to be associated with a significant risk of infection since they do not lead to a state of meaningful immunosuppression. Conversely, patients treated with second-line drugs show a contradiction in the results [59].

In the management of DMT in MS patients during the COVID-19 pandemic, the International Multiple Sclerosis Federation recommends continuing the treatment together with general measures of social isolation and special monitoring of lymphocyte counts for those undergoing treatments with S1P receptor modulators, dimethylfumarate, or teriflunomide [60,61,62]. For example, it is not recommended to modify the frequency or dose of treatment with natalizumab, considering that the risk of reactivation may be greater than the risk of contracting the viral infection, although it is possible to consider administration in prolonged intervals. In cases of DMT with the anti-CD20 monoclonal antibody, it is recommended to delay the doses until the risk of COVID-19 infection is reduced [62] and to use rituximab. In addition, in cases of severe relapse with hospitalization, the use of intravenous methylprednisolone is recommended together with the use of plasmapheresis as an alternate intervention [60,61].

In MS, the greatest infection risk has been suggested for highly effective treatments in the management of MS due to their marked effect on the lymphocyte population, for instance, alentuzumab or cladribine, primarily due to lymphopenia, which has been described as being associated with COVID-19 [60]. Finally, the authors argued that the potential beneficial effect of DMT in MS is based on the downregulation of pro-inflammatory cytokines and the reduction of immune responses through interferons-beta, IL-1beta, IL-6, and TNF-alfa. Glatiramer acetate could be responsible for shifting from a pro-inflammatory to an anti-inflammatory state and for blocking IFN-mediated activation of macrophages. Leflunomide, the active metabolite of teriflunomide, is able to downregulate pro-inflammatory cytokines IL-1, IL-6, and TNF-alfa released from overactivated macrophages [59,60].

A paper was published by Machado and cols. In this paper, the authors suggested the occurrence of a major risk of long COVID in adults with a history of obstructive sleep apnea, described the nervous system damage, and recommended steps to manage this syndrome in COVID-19 patients [63].

Another two papers were published on this topic by Cuban authors, who addressed paraclinical intervention and COVID-19.

One, published by Morales Chacón [55], reviewed the available evidence on the role of electroencephalogram to evidence the impact of infection by SARS-CoV-2 on the CNS. The study was based on an exhaustive review of the publications indexed in the National Library of Medicine of the United States of America (PubMed/Medline), emphasizing reports of epileptic seizures, non-seizure epileptic states, acute symptomatic seizures, and encephalopathies, as well as the main recommendations for EEG recording and interpretation for patients being treated for COVID-19 with both outpatient and hospitalization status. At the same time, the authors emphasized that intensive care units can conclude that EEG provides relevant information for the evaluation of neurological manifestations in patients being treated for COVID-19 and why carrying out different types of EEG studies is required to assess the risk versus benefit, in addition to demanding compliance with biosafety protocols [64].

The second article was published by Valdés Sedeño and Vega Treto [65]. The article took into account the clinical particularities of COVID-19 that affect the CNS. Clinical neurophysiologist services could be required for several studies, and it was recognized that this diagnostic tool is useful for monitoring patients in intensive care units. The authors also approved of neurophysiology personnel needing to know and apply protective measures to protect themselves and patients. In the future, these paraclinical measures must continue to be improved to mitigate the real impact of the pandemic’s effect on the nervous system.

## 4. Discussion and Conclusions

During the recent pandemic, interesting data were published by Cuban authors on translational approaches to COVID -19, showing a growing interest in this complex disease and brain function. Exciting news in several aspects of SARS-CoV-2 infection and CNS emerged, included new insights into psychology, disease pathogenesis focused on the brain invasion mechanisms, neuroimmunology, and neuropsychiatric manifestation, as well as some advice on treatment strategies for COVID-19 itself as a disease derived from the pandemic or in susceptible people with a history of neurological autoimmune disorders, which are all summarized in this paper. In all cases, these experiences became relevant to our country and our region, and although it is recognized that some aspects would benefit from more in-depth research, like those related to stroke or other neurological sequels early on or in the long term, the results of the reported papers are relevant.

This review focused on COVID-19 and the nervous system from a Cuban perspective. Cognitive and affective emotional states, pathogenesis, biomarkers, clinical manifestations, and intervention strategies were addressed. The studies that have been shown in this article on the world’s confrontation with COVID-19, and Cuba’s response to the pandemic, were fundamentally successful. Cuban science has a coherent and systematic scientific policy that exhibits solid results and has international recognition.

By the way, it is necessary to continue to release longitudinal investigations in all directions around the world so that the scientific community can not only define more precise mechanisms to explain neurological manifestations post-COVID-19 but also find more early multidisciplinary interventions to prevent neurological, neuropsychological, and neuropsychiatric sequels in the long term and provide more effective neuroprotection against brain infection in the context of neurotrophic virus infection. Based on these reports, we also suggest that it is necessary for cognitive and neuropsychological function to be monitored in all surviving COVID-19 patients.

## Data Availability

No new data were created or analyzed in this study. Data sharing is not applicable to this article.
